# Improving the Completeness of Chromosome-Level Assembly by Recalling Sequences from Lost Contigs

**DOI:** 10.3390/genes14101926

**Published:** 2023-10-10

**Authors:** Junyang Liu, Fang Liu, Weihua Pan

**Affiliations:** 1 Zhengzhou Research Base, State Key Laboratory of Cotton Biology, School of Agricultural Sciences, Zhengzhou University, Zhengzhou 450001, China; liujun_yang@gs.zzu.edu.cn; 2 Shenzhen Branch, Guangdong Laboratory of Lingnan Modern Agriculture, Genome Analysis Laboratory of the Ministry of Agriculture and Rural Affairs, Agricultural Genomics Institute at Shenzhen, Chinese Academy of Agricultural Sciences (ICR, CAAS), Shenzhen 518120, China; 3 National Key Laboratory of Cotton Bio-Breeding and Integrated Utilization, Institute of Cotton Research, Chinese Academy of Agricultural Sciences (ICR, CAAS), Anyang 455000, China

**Keywords:** genome assembly, gap free, single-copy gene, completeness

## Abstract

For a long time, the construction of complete reference genomes for complex eukaryotic genomes has been hindered by the limitations of sequencing technologies. Recently, the Pacific Biosciences (PacBio) HiFi data and Oxford Nanopore Technologies (ONT) Ultra-Long data, leveraging their respective advantages in accuracy and length, have provided an opportunity for generating complete chromosome sequences. Nevertheless, for the majority of genomes, the chromosome-level assemblies generated using existing methods still miss a high proportion of sequences due to losing small contigs in the step of assembly and scaffolding. To address this shortcoming, in this paper, we propose a novel method that is able to identify and fill the gaps in the chromosome-level assembly by recalling the sequences in the lost small contigs. Experimental results on both real and simulated datasets demonstrate that this method is able to improve the completeness of the chromosome-level assembly.

## 1. Introduction

De novo genome assembly is a vital fundamental technique in genomics and computational biology. For a long time, the construction of complete reference genomes for complex eukaryotic genomes has been hindered by the limitations of sequencing technologies, including sequencing errors, uneven sequencing coverage, and chimeric reads [1,2]. The incompleteness of reference genome sequences poses limitations in comprehending genetic variations and functionality in the genomes of humans, animals, and plants, hampering research and treatment endeavors related to cancer, infertility, and various genetic disorders. Additionally, it can lead to data contamination and the erroneous identification of variations during specific data analyses [3,4].

The advancements in novel sequencing technologies have greatly enhanced the completeness of genome assembly. Pacific Biosciences (PacBio) HiFi data and Oxford Nanopore Technologies (ONT) Ultra-Long data, leveraging their respective advantages in accuracy and length, have the capability to generate complete chromosome sequences [5,6,7]. The Telomere-to-Telomere (T2T) consortium published a collection of articles in 2022, showcasing the latest human reference genome. This significant breakthrough involved the seamless assembly of all autosomes and the X chromosome, except for the Y chromosome, overcoming unresolved challenges that accounted for 8% of the Human Genome Project [8]. The successful completion of this long-term endeavor represents a major milestone in human genome sequencing, reflecting the culmination of nearly four decades of dedicated global scientific efforts [8,9,10]. Rice played a pioneering role in the development of Telomere-to-Telomere (T2T) genomes. In June 2021, two rice T2T genomes were published, establishing the first complete reference genomes in plant species. This achievement marked a significant milestone in rice genomics, following the earlier releases of rice genome maps in 2002 and 2005. Furthermore, in June 2022, the T2T genomes of four backbone parents of hybrid rice were published, indicating the emergence of the T2T era in heterosis research and hybrid breeding [11]. In November 2021, the T2T genome of Arabidopsis thaliana Col-CEN was published, providing a comprehensive assembly that includes the centromeric regions. Similarly, in June 2022, the T2T genome of Arabidopsis thaliana Col-PEK was published, representing the complete assembly of the Arabidopsis thaliana genome to date [12].

In the field of methodology, numerous algorithms have been developed for de novo assembly, including hifiasm [13], verkko [14], hicanu [15], and flye [16]. These algorithms incorporate unique core ideas and exhibit distinct advantages. Hifiasm focuses on accurately resolving overlaps between long reads to produce highly contiguous assemblies, particularly suited for repetitive regions. Verkko integrates accurate long and noisy ultra-long sequencing reads, employing a population-aware approach to capture genetic variations and improve assembly quality. Hicanu combines long and short reads, leveraging the former to span repetitive regions and resolve complex structures, while the latter provides precise sequence information, resulting in high-quality assemblies. Flye is specifically designed for highly repetitive genomes, utilizing an iterative approach to resolve repeats and generate highly contiguous assemblies for challenging genomic regions.

Nevertheless, for the majority of genomes, it remains challenging to generate complete chromosome sequences. For example, even in the highest-quality diploid human genome (HG002), there are still 195 gaps, and more importantly, missing sequences persist in gap-free regions [17]. This is primarily because assembly tools in complex genomic regions often use simplified methods that overlook specific gaps in order to produce longer, contiguous contigs, resulting in the formation of smaller contig sequences within these gaps [13]. Overall, there is an urgent need for a method to further improve the completeness of the chromosome-level assemblies.

To address this challenge, we propose a novel method that is able to identify and fill the gaps in the chromosome-level assembly using the sequences in the small contigs lost. To achieve this goal, we use single-copy genes as markers to identify these missing sequences in contigs. To increase the possibility that the sequences missing in chromosome-level assembly appear in the contigs, our method allows the contigs set to include multiple assemblies generated by different assemblers and different parameters. We tested our method on both real and simulated data, and the results demonstrated that our approach can further improve completeness to a certain extent on high-quality chromosome-level assembly.

## 2. Materials and Methods

We obtain HiFi reads of Arabidopsis from the study by Hou et al. [12], with the Genome Sequence Archive (GSA) accession number CRA005381. The data used for Arabidopsis are from a homozygous diploid, where the differences between the two haplotypes are minimal, allowing it to be treated as a haploid. For watermelon, the HiFi reads are provided by Deng et al. [18] and accessed from National Genomics Data Center (NGDC), under the BioProject accession number PRJCA008083. HiFi reads of potato are obtained from Zhou et al. [19], and the corresponding Sequence Read Archive (SRA) accession number in the NCBI database is SRR11318516. These reads are part of “OptiSpud Project”, which aims to revolutionize potato breeding by transitioning from tetraploidy to diploidy and hybrid seeds [19]. We utilize diploid potato material from this project, representing the diploid state of the potato genome, for our experiments.

**Figure 1:**
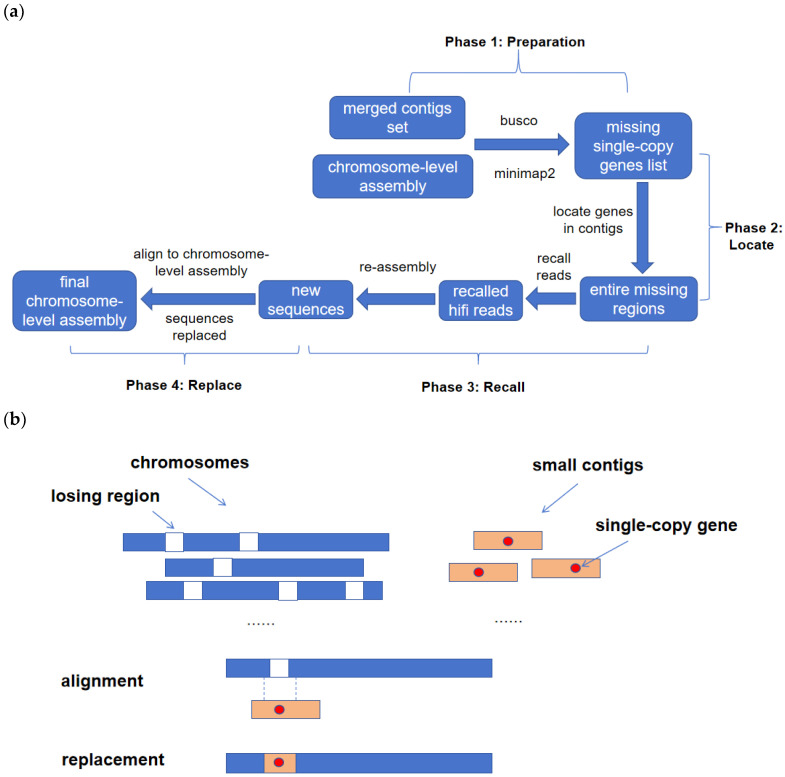
(**a**) Algorithm flowchart; the input to the algorithm is a chromosome-level assembly and a merged set of contigs. (**b**) Sequence replacement example; the blue elongated bars represent multiple chromosomes of chromosome-level assembly results, where white squares represent missing regions, yellow small squares are small contigs, and red circles are single-copy genes.

The proposed filling method is an alignment-based algorithm that takes a chromosome-level assembly and a merged set of contigs from multiple assemblies generated using different tools and parameters as inputs. The algorithm comprises four phases. In the first phase, the completeness of the initial assembly results is assessed using BUSCO [20], and a list of single-copy genes missing in the chromosome-level assembly but appearing in contigs is identified. The second phase locates these missing single-copy genes in contigs and identifies the entire missing regions. In the third phase, the HiFi reads related to the missing genomic regions are recalled, and these reads are then input into an assembly tool to regenerate new sequences. In the fourth phase, the newly obtained sequences are aligned one by one with the chromosome-level assembly, gradually increasing the level of completeness. The pipeline of the algorithm is illustrated in Figure 1.

### 2.1. Phase 1: Preparation

At a high level, phase one has two major steps. In step 1, we separately perform preliminary BUSCO evaluations on the merged contigs set and chromosome-level assembly using the embryophyta_odb10 database of BUSCO with default parameters. Based on the BUSCO results, we obtain separate lists of missing single-copy genes for both merged contigs set and chromosome-level assembly. By taking the set difference between the two lists, we derive the merged contigs set’s list of single-copy genes that can be used to fill the chromosome-level assembly. In the second step, we perform alignments using minimap2 with the parameter “-x asm5”. This includes aligning the merged contigs set with chromosome-level assembly, aligning the merged contigs set with HiFi reads, and aligning chromosome-level assembly with HiFi reads.

### 2.2. Phase 2: Locate

In this phase, our objective is to determine the precise positions of the missing single-copy genes identified in the first phase. The initial BUSCO evaluations on the merged contigs set provide us with detailed information on the location of each complete single-copy gene, including the specific sequence it belongs to and its exact position within that sequence. Utilizing this information, along with the alignment results obtained between merged contigs set and chromosome-level assembly during the first phase, we filter the alignment information to identify the positions that contain the missing single-copy genes. To completely recall reads in the following step, we define here the whole contig containing at least one missing single-copy gene as the missing genomic region.

### 2.3. Phase 3: Recall

During this phase, the primary objective is to recall HiFi reads and assemble them into new sequences based on alignment information. The initial step involves obtaining HiFi reads associated with the missing genomic regions. To completely obtain reads, we not only recall reads aligned to the related contigs but also the neighboring genomic regions in the chromosome-level assembly separately. Next, the two sets of recalled HiFi reads are merged into a unified set and utilized as input for assembly tools such as hifiasm or hiCanu, with default parameters, to generate new sequences. Since the recalled reads at a genomic region may be assembled into multiple sequences, in this step, the correct sequence among them is selected according to the integral appearance of this single-copy gene. More specifically, we evaluate the integrity of the current single-copy gene on all these sequences by subjecting them to BUSCO using the embryophyta_odb10 database and default parameters. A successful recall is indicated if the single-copy gene is determined to be intact. Finally, the sequences containing the single-copy genes are extracted and stored as a distinct sequence file. This file is subsequently used for further analysis and processing.

### 2.4. Phase 4: Replace

In the final phase, we align the assembled new sequences to the chromosome-level assembly, adhering to the sequential order of the missing single-copy genes. According to the alignment positions, we replace the corresponding sequences on the chromosomes by new sequences. During this process, we diligently validate the presence of the missing genes within the designated alignment positions. The replacement is exclusively carried out once the presence of the missing genes is conclusively ascertained within the alignment positions. After the sequence replacement is completed, we conduct a BUSCO analysis using the embryophyta_odb10 database and default parameters. This analysis aims to evaluate the integrity of the final chromosomes and ensure there is no decrease in the number of single-copy genes.

## 3. Results

To evaluate the performance of the algorithm, we conducted experiments using both real and simulated data. We tested multiple species, including Arabidopsis (*Arabidopsis thaliana*), Watermelon (*Citrullus lanatus*), Rice (*Oryza sativa*), and Potato (*Solanum tuberosum*). Arabidopsis, with its small genome size of approximately 125 Mb, serves as an ideal model organism for studying plant genetics and molecular biology. Watermelon, a valuable cash crop with a genome size of around 425 Mb, is crucial for understanding agronomic traits and disease resistance. Rice, a vital staple food with a genome size of approximately 430 Mb, is essential for unraveling the genetic functions of food crops. Potato, an important staple crop, utilizes genetic information to enhance disease resistance, adaptability, and yield.

After obtaining the HiFi reads, contig-level assembly results were generated using mainstream assembly tools, namely Hifiasm, Flye, and HiCanu, employing default parameters. The resulting contig-level assemblies were merged to form a comprehensive collection of contigs, referred to as “merged contigs set”. The completeness of the assembly was assessed using the embryophyta_odb10 database of BUSCO, utilizing default parameters. Based on the BUSCO evaluation, the contig-level assembly with the highest number of missing single-copy genes was selected. Subsequently, RAGTAG was employed in scaffold mode to generate chromosome-level assembly results, referred to as chromosome-level assembly.

Simultaneously, we employed the simulation software pbsim3 [21] to generate simulated HiFi reads. It is important to note that pbsim3 does not directly simulate HiFi reads but instead simulates the generation of CLR (circular consensus reads) through multi-pass sequencing using the parameter “--strategy wgs --method qshmm --depth 20 --pass num 10”. The output of the PBSIM simulation, in SAM format, was converted to BAM format data and subsequently input into the CCS software, which generated the HiFi reads.

After obtaining simulated HiFi reads, the same operations were performed as with real data to generate a merged contigs set and chromosome-level assembly. Based on the simulation haploid genome, the script SimSID.py by Zhang et al. [22] was used to artificially construct a diploid genome by introducing different numbers of SNPs, insertions, and deletions using various parameters. The default parameters were used for the composition of haplotype 1 (hap1), while the composition parameters for haplotype 2 (hap2) were set as “-s 0.02 -i 0.02 --insert_length 12 -d 0.02 --delete_length 12”. Multiple sets of simulated data were generated and named as sim_Rice_1, sim_Rice_2, and sim_Rice_dip. Among them, sim_Rice_1 and sim_Rice_2 represented haploid datasets, while sim_Rice_dip represented a diploid dataset.

There were two types of datasets used for algorithm testing: real datasets and simulation datasets, each consisting of haploids and diploids. In the real datasets, Arabidopsis and watermelon were haploid, while potato was diploid. In the simulated datasets, sim_Rice_1 and sim_Rice_2 were haploid, while sim_Rice_dip was diploid. After executing the algorithm, the number of missing single-copy genes decreased, resulting in an improvement in the completeness of the chromosome-level assembly results.

**Figure 2:**
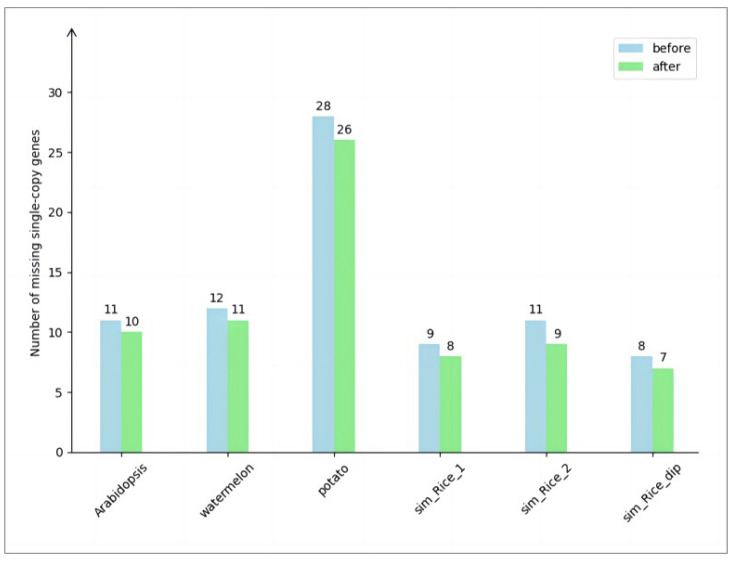
Comparison of missing number of single-copy genes. The horizontal axis of the chart represents the names of the experimental datasets, while the vertical axis denotes the number of missing single-copy genes. The numerical values displayed on the bar chart indicate the count of missing single-copy genes. The light-blue bars represent the number of single-copy gene losses before executing the algorithm, while the light-green bars represent the number of single-copy gene losses after executing the algorithm.

The experimental results are shown in Figure 2. In the real haploid dataset, the initial chromosome-level assembly of Arabidopsis had 11 missing single-copy genes. After applying the algorithm, 10 genes remained missing and 1 gene was successfully recovered. Similarly, in the chromosome-level assembly of watermelon, initially, 12 single-copy genes were missing. After the algorithm execution, 11 genes were still missing and 1 gene was successfully recovered.

In the real diploid dataset, the chromosome-level assembly of potato initially resulted in the loss of 28 single-copy genes. After executing the algorithm, 26 genes remained missing, while 2 single-copy genes were successfully recovered.

In the simulated data, the initial chromosome-level assembly of haploid rice sim_Rice_1 showed a loss of 9 single-copy genes. After applying the algorithm, 8 genes remained missing and 1 gene was successfully recovered. Similarly, for haploid rice sim_Rice_2, the initial assembly exhibited a loss of 11 single-copy genes. After executing the algorithm, 9 genes remained missing and 2 genes were successfully recovered. In the case of diploid rice sim_Rice_dip, the initial assembly showed a loss of 8 single-copy genes. After applying the algorithm, 7 genes remained missing and 1 gene was successfully recovered.

To conclude, our algorithm exhibits effectiveness across various datasets, encompassing real and simulated data, as well as haploid and diploid datasets.

## 4. Conclusions and Discussion

In this paper, we present a new method for improving the completeness of chromosome-level assembly by recalling the sequences in the small contigs lost in the step of assembly and scaffolding. To achieve this goal, we use single-copy genes as markers to identify these missing sequences in contigs. To increase the possibility that the sequences missing in chromosome-level assembly appear in the contigs, our method allows the contigs set to include multiple assemblies generated by different assemblers and different parameters. Experimental results on both real and simulated datasets demonstrate that this method is able to improve the completeness of the chromosome-level assembly to some extent.

In the future, the method can be further improved in effectiveness. In the current version of the method, we only fill the gaps with the single-copy gene contained in the alignments between new sequences and chromosome-level assembly. But in some situations, the single-copy gene is out of the aligned genomic region, which results in the incompleteness left in the gap-filled chromosome-level assembly. Another limitation is that if there are missing genomic regions that do not include single-copy genes, then our method is not applicable. In next version of the method, we will try to fill this kind of gap by replacing larger sequences with multiple alignments.

## Data Availability

HiFi reads of Arabidopsis with the Genome Sequence Archive (GSA) accession number CRA005381. For watermelon, the HiFi reads were accessed from the National Genomics Data Center (NGDC) under the BioProject accession number PRJCA008083. HiFi reads of potato have the corresponding Sequence Read Archive (SRA) accession number in the NCBI database, which is SRR11318516.

## Code Availability

https://github.com/panlab-bio/Icca (accessed on 20 September 2023).
